# Tubo-Ovarian Abscesses: Epidemiology and Predictors for Failed Response to Medical Management in an Asian Population

**DOI:** 10.1155/2019/4161394

**Published:** 2019-06-02

**Authors:** Grace Ming Fen Chan, Yoke Fai Fong, Kai Lyn Ng

**Affiliations:** Department of Obstetrics and Gynaecology, National University Hospital Singapore, Singapore

## Abstract

Pelvic inflammatory disease (PID) complicated by tubo-ovarian abscesses (TOA) has long-term sequelae in women of reproductive age. Consensus on the optimal treatment of TOA remains lacking. Most clinicians utilize antibiotics as a first-line conservative approach, failing which invasive intervention is adopted. Our aim is to identify risk factors predicting failed response to conservative medical management for TOA in an Asian population. A retrospective cohort study of 136 patients admitted to a tertiary hospital in Singapore for TOA between July 2013 and December 2017 was performed. Patients were classified into 2 groups: successful medical treatment with intravenous antibiotics and failed medical treatment requiring invasive intervention. 111 (81.6%) of patients were successfully treated with conservative medical approach using intravenous antibiotics; 25 (18.4%) required invasive intervention having failed medical therapy. Multivariate logistic regression model adjusted for age, ethnicity, C-reactive Protein (CRP), TOA size, and body mass index (BMI) showed the odds ratio (OR) of each centimetre increase in TOA size to be 1.28 (95% confidence interval (CI) 1.03-1.61;* P*=0.030) and every kg/m2 increase in BMI to be 1.10 (95% CI 1.00-1.21;* P*=0.040). Failed medical management was predicted by a cutoff of TOA size ≥ 7.4 cm and ≥ BMI 24.9 kg/m2. Patients who failed medical treatment received a mean of 4.0±2.1 days of antibiotics before a decision for invasive intervention was made, with a significantly longer intravenous antibiotic duration (9.4±4.3 versus 3.6±2.2 days;* P *<0.001) and prolonged hospitalization (10.8± 3.6 versus 4.5 ± 2.0 days;* P* <0.001) compared to the medical group. Patients with higher BMI and larger TOA size were associated with failed response to conservative medical management in our study population. Early identification of these patients for failed medical therapy is imperative for timely invasive intervention to avoid prolonged hospitalization, antibiotic usage, and patient morbidity.

## 1. Introduction

Pelvic inflammatory disease (PID) is an ascending upper genital tract infection resulting in endometritis ans salpingitis and, in severe cases, tubo-ovarian abscesses (TOA). TOA is an inflammatory mass involving the ovary, fallopian tube, and surrounding pelvic organs. It is a serious complication of PID, affecting 10-15% of patients [1]. Risk factors for TOA are similar to PID—multiple sexual partners, previous history of PID, pre-existing sexually transmitted diseases (STDs), immunosuppression, or endometriosis [2]. Older models of intrauterine contraceptive device (IUCD) with multifilament strings have also been associated with TOAs. [3, 4]. Patients are commonly in the reproductive age group and are sexually active [2]. Typical presenting complaints include fever, abdominal pain, and foul-smelling vaginal discharge. On examination, lower abdominal tenderness, cervical excitation, and adnexal tenderness may be present, with raised inflammatory markers [5]. TOA is potentially life-threatening if it ruptures or leads to severe sepsis. Delayed treatment could result in long-term sequelae in affected women, such as chronic pelvic pain, recurrent PID, distorted pelvic anatomy, infertility, and ectopic pregnancies [6, 7].

International consensus on the optimal treatment algorithm of TOA remains lacking. Majority of clinicians utilize a conservative approach with empirical broad spectrum antibiotics as first-line management for aerobic and anaerobic cover [2, 8, 9]. However, it is known that about 25-30% of patients fail to respond to medical treatment and go on to require invasive intervention [1, 8, 10]. Surgical options for source control include laparotomy or laparoscopic abscess drainage, salpingectomy, or adenexectomy [2]. Alternatively, radiologically guided percutaneous drainage has become increasingly popular with decreased morbidity compared to conventional surgery [11, 12]. The optimal selection and timing for invasive treatment in patients with TOA are unclear.

Nonidentification of patients at increased risk of unsuccessful conservative medical management with empirical antibiotics early in the PID clinical course limits the clinicians' ability to individualize treatment. There have been some studies in literature that have identified risk factors for antibiotic treatment failure. In the USA, Farid et al. described TOA size and high total white (TW) count as risk factors [13], while Greenstein et al. found age and parity along with TOA size and high TW count to be significant risk factors [14]. Güngördük et al. studied TOA patients in Western Turkey and found that TOA diameter, erythrocyte sedimentation rate (ESR), and C-reactive protein (CRP) levels were predictive of patients who required invasive treatment [15]. However, there remains a paucity of studies in the Asian population. Hence, the aim of our retrospective review is to identify risk factors predicting failed response to conservative medical treatment for TOA in the multiethnic Asian population of Singapore.

## 2. Material and Methods

**Figure 1:**
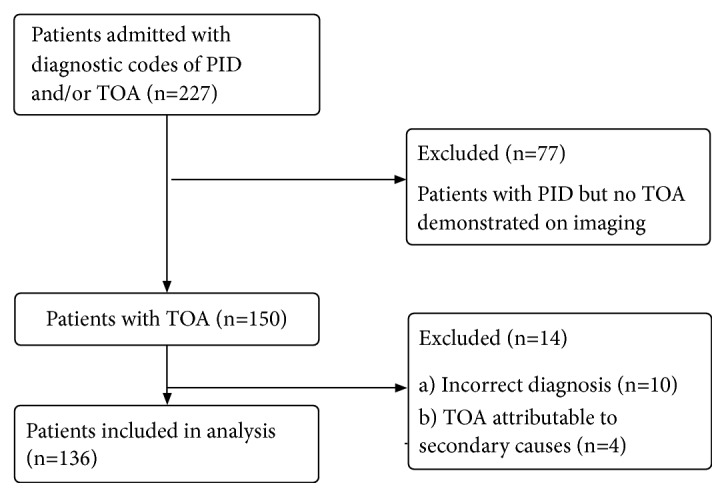
Flowchart of patients included in this study.

A retrospective cohort study was performed on a total of 136 patients admitted to a tertiary hospital in Singapore for PID complicated by TOA between July 2013 and December 2017. Patients were retrospectively identified through a search of the hospital's electronic database, using diagnostic codes for PID and/or TOA. Those who had clinical signs and symptoms of primary PID as per the British Association for Sexual health and HIV (BASHH) guidelines [16] as well as confirmation of TOA on radiological imaging were included. As per BASHH, we included patients who presented with symptoms of fever, lower abdominal pain, dyspareunia, abnormal vaginal bleeding, secondary dysmenorrhea, and/or abnormal vaginal discharge and displayed pyrexia, lower abdominal tenderness, adnexal tenderness, cervical motion tenderness, and/or purulent vaginal discharge on physical examination [16]. Patients who had TOA attributable to secondary causes (e.g., perforated appendicitis, diverticulitis, and systemic infections such as tuberculosis) were excluded. There were no patients who were immunocompromised or had underlying gynaecological malignancy in our cohort. 227 patients' charts were reviewed to confirm eligibility. 77 patients were excluded as they were admitted for PID without any tubo- ovarian abscess on imaging. 14 patients were excluded due to incorrect diagnosis on admission or TOA attributable to secondary causes. 136 patients were eventually included in our study for analysis (Figure 1).

Following approval from the institutional ethics committee, data from the hospital's electronic database on demographics, clinical factors, investigation parameters and patient outcomes of PID and TOA admissions were extracted. Patients' records were reviewed for the following signs and symptoms of PID on presentation: lower abdominal pain, fever, abnormal vaginal discharge, rebound or guarding on abdominal examination, and presence of adnexal tenderness or cervical motion excitation. Fever was defined as a temperature ≥38 degree Celsius. All patients had confirmation of TOA by radiological imaging (either via pelvic ultrasound and/or computed tomography (CT) scan). In cases where bilateral TOAs were found, the size of the larger TOA was used for analysis. Other investigations that were studied included TW count, CRP, and endocervical swabs for Neisseria Gonorrhea and Chlamydia trachomatis.

Patients were classified into 2 groups: (1) successful medical treatment with intravenous antibiotics and (2) failed medical therapy requiring invasive intervention. The primary outcome was failed response to medical therapy requiring invasive intervention. All patients were started on first-line inpatient antibiotic regime on admission as per BASHH recommendations [16]: intravenous Ceftriaxone, Metronidazole, and oral Doxycycline; those with documented allergies were started on intravenous Clindamycin with Gentamicin or intravenous Ciproflocaxin with Metronidazole. Group 1 consisted of patients who exhibited good clinical response within 48 to 72 hours of initiating antibiotic therapy, with resolution of their presenting complaints (e.g., abdominal pain and fever) and downtrending inflammatory markers. Conversely, Group 2 consisted of patients who had persistent abdominal pain, pyrexia, and/or rising inflammatory markers despite an adequate trial of intravenous antibiotics. Decision for the type of invasive intervention was based on individual clinician discretion and included laparotomy/laparoscopic or radiological guided abscess drainage, salpingectomy, or adnexectomy. In addition, records were reviewed to see if patients received counselling on opportunistic sexually transmitted diseases (STD) screen, importance of barrier methods in preventing PID, contact tracing, and had scheduled follow up in outpatient clinics.

### 2.1. Statistical Analysis

Data was analyzed using IBM SPSS Statistics version 20 (SPSS Inc., Chicago, IL, USA). Test of normality of distribution of variables was done with Shapiro-Wilk test. The Chi-square test was used to analyze categorical variables. For continuous variables, the t test was used to analyze normally-distributed variables, and the Mann–Whitney U test was used for variables that did not have a normal distribution. Receiver operating characteristic (ROC) curves were used to evaluate for sensitivity, specificity, and optimum cutoff values for risk factors at predicting surgical management. Multiple logistic regression was used to explore predictive factors of risk factors that were shown on univariate analysis to be significant for surgical treatment, adjusting for age and ethnicity. A p-value of <0.05 was considered to be statistically significant.

Ethical approval was obtained from National Healthcare Group's (NHG) Domain Specific Review Board (DSRB). DSRB reference number was 2016/00835, and approval was obtained in March 2018.

## 3. Results

A total of 136 patients were included in our retrospective review, with the mean age of 37.7 ± 9.7 years. 46 (33.8%) patients were Chinese, 48 (35.3%) were Malay, 16 (11.8%) were Indian, and 26 (19.1%) consisted of other ethnicities. 111 (81.6%) patients were treated successfully with antibiotics, while 25 (18.4%) patients failed medical treatment and required surgical intervention.

Demographic characteristics of the patients in both groups are presented in Table 1. Patients in Group 2 tended to be older (39.9 ± 9.0 years versus 37.2 ± 9.8 years), although this was not statistically significant (*P*=0.209). They also had a significantly higher BMI compared to patients in Group 1 (30.6 ± 12.4 versus 25.5 ± 5.4 kg/m^2^) on univariate analysis (*P*=0.011). Clinical and biochemical laboratory values on admission between the two groups were compared and presented in Table 2. Univariate analysis showed that patients in Group 2 were more likely to present with pyrexia ≥ 38 degrees Celsius (88.0% versus 39.6%;* P*<0.001), higher CRP (183.7 ± 83.5 versus 151.7 ± 112.7;* P* = 0.050), and larger TOA size (8.2 ± 2.7 versus 6.9 ± 3.6* P* =0.002).

**Figure 2:**
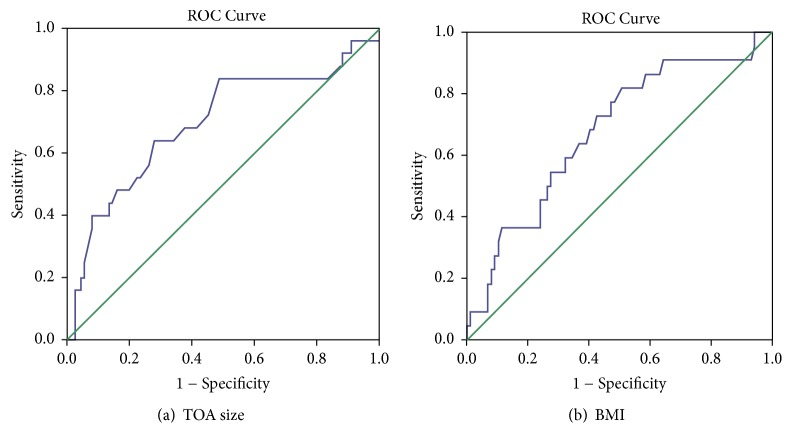
ROC curves to assess the usefulness of (a) TOA size and (b) BMI to predict failure of medical treatment with antibiotics in patients with TOA.

Multivariate logistic regression model adjusting for age, ethnicity, CRP, BMI, and TOA size is shown in Table 3. Although fever on presentation was a significant risk factor for failure of medical management, it was not included in the final model due to high multicollinearity. BMI and TOA size were found to be independent predictors of failure of medical management, with the odds ratio (OR) of each cm increase in TOA size to be 1.28 (95% confidence interval (CI) 1.03-1.61;* P*=0.030) and every kg/m^2^ increase in BMI to be 1.10 (95% CI 1.00-1.21;* P*=0.040). ROC curve analysis to assess for the usefulness of TOA size and BMI to predict the failure of TOA treatment with antibiotics is shown in Figure 2. ROC curves showed that the need for invasive intervention could be predicted by a cutoff TOA size of 7.4 cm (area under the curve (AUC) 0.70, sensitivity 64.0%, and specificity 72.1%,* P*= 0.002) and BMI 24.9 kg/m^2^ (AUC 0.68, sensitivity 72.7%, and specificity 57.5%,* P*= 0.011), respectively.

Comparison of patient outcomes between the two groups is presented in Table 4. Patients in Group 2 had a significantly prolonged inpatient stay as compared to patients in Group 1 (10.8 ± 3.6 versus 4.5 ± 2.0 days;* P*<0.001), with a longer duration of intravenous antibiotics (9.4 ± 4.3 versus 3.6 ± 2.2 days;* P*<0.001). Amongst the 25 patients in Group 2 who required invasive intervention, 17 (68%) had abscess drainage, 7 (28%) had salpingectomy, and 1 (4%) had salpingo-oophrectomy. Minimally invasive was the predominant approach, with 17 (68%) patients undergoing laparoscopy, 5 (20%) radiological guided percutaneous, and the remaining 3 (12%) having laparotomies. These patients had a trial of antibiotics for a mean of 4.0 ± 2.1 days before a decision for invasive intervention was made.

In our retrospective review, 29.4% of patients had been counselled for and underwent opportunistic STD screen, 27.9% received counselling on the importance of barrier methods, and 17.6% had partner contact tracing performed. All patients were scheduled for a followup visit in the outpatient clinic within 2 weeks of discharge, of which the majority (86.8%) complied with, while the remaining 13.2% defaulted.

## 4. Discussion

Existing literature [1, 8, 10] shows that approximately 70% of patients with TOA respond to conservative medical management with broad spectrum antibiotics, with the rest requiring invasive intervention. This is consistent with the results of our study, where 81.6% of patients were successfully treated with antibiotic therapy. TOA size has been described as a predictor of failed medical treatment in various published studies, likely due to the decreased ability of antibiotics to penetrate larger abscess cavities. Dewitt et al. [17] reported a 43% failure rate of conservative treatment for patients with TOA larger than 8cm. Topçu et al. [18] found that TOA size greater than 6cm increased the risk of surgery and hospitalization stay. Reed at al. [9] demonstrated an increase in treatment failure with increasing TOA size, with 35% of patients having TOA size 7 to 9 cm and nearly 60% of patients having TOA size greater than 10cm failing conservative treatment. Our retrospective review also identified TOA size as an independent risk factor that predicts failure of conservative medical management in patients with PID complicated by TOA. In particular, a cutoff TOA size of more than 7.4 cm was demonstrated in our study, with every 1 cm increase in size associated with a 1.28 times increased risk for invasive intervention. We also found that high grade pyrexia ≥38 degree Celsius on admission was significantly associated with failure of medical management requiring invasive intervention—this is consistent with Kinay et al. [19] who reported fever on admission as an independent predictor of patients requiring surgical treatment.

Another novel independent risk factor that emerged in our study was BMI—this has not been well described in existing literature. Our analysis showed that patients with a BMI ≥24.9 kg/m^2^ were at higher risk of medical treatment failure, with a 1.10 times increased risk of requiring invasive intervention for every kg/m^2^ increase. In an Asian population, a BMI of ≥24.9 kg/m^2^ indicates overweight going on to obesity according to the obesity classification according to World Health Organization (WHO) and Asia-Pacific guidelines [20]. Obesity has been reported to reduce immunological response by the production of proinflammatory factors by adipose tissue and altered T-cell function [21, 22]. Furthermore, increased weight and body fat affect the pharmacokinetics and pharmacodynamics of antibiotics, thus resulting in subtherapeutic tissue concentration [23].

Unlike existing studies, we did not find a significant association between failure of medical management and age, parity, and TW counts. Both Terao et al. [24] and Kuo et al. [25] identified CRP levels as an important prognostic factor for surgery; while univariate analysis for our study also showed CRP to be similarly associated with medical treatment failure, this was no longer significant after adjusting for cofounders, possibly due to our limited numbers.

Our study observed a significant longer duration of both antibiotics use and hospitalization stay for patients that failed medical treatment. Patients in Group 2 had an average of 4 days of intravenous antibiotics before medical treatment failure was recognised and decision for invasive treatment was made. This resulted in prolonged length of stay and increased costs. Hence, the findings of our study are important to identify patients at risk for antibiotic failure early on so as to guide decision making for timely invasive treatment. Goharkhay et al. demonstrated that patients with TOA that underwent primary drainage had shorter hospitalization stay and more rapid resolution of fever as compared to patients that were treated with antibiotics alone [26].

We did not specifically compare patient outcomes amongst those who underwent surgical or radiological approach in our study. Growing evidence has shown ultrasound or CT-guided drainage to be as effective as surgical drainage for the treatment of TOA without associated risks of general anaesthesia and surgery. Gjelland et al. showed a success rate of 93.4% in transvaginal ultrasound-guided aspiration with no procedure-related complication amongst 302 women with TOA [27]. An area of potential future research could be to compare the success rates between different modes of invasive treatment.

Our limitations were that of being a retrospective cohort study with relatively small sample size, where data was based on hospital electronic records. There was potential selection bias, with the decision for invasive intervention driven by individual clinicians based on personal clinical judgement and experience rather than protocol-based, although heterogeneity was limited as decision making was largely confined to a few clinicians within the department. We did not specifically investigate the role of diabetes in our patient treatment outcomes, which may have been a possible confounder in the relationship between BMI and medical treatment failure.

A notable strength of our retrospective review is that it is the first in South-east Asia to examine risk factors for antibiotic treatment failure in patients with PID complicated by TOA across a multiethnic population. It is also one of the few clinical studies to have examined BMI specifically as a risk factor. The low rates of patient counselling for opportunistic STD screen and barrier method usage as well as partner contact tracing revealed in our study has since led to increased awareness and resulted in a departmental workflow spearheaded by the authors with increased nurses' empowerment to optimise the adjunct management of these patients with PID and/or TOA.

## 5. Conclusion

Our study provides a valuable clinical insight in predicting failed response to conservative medical management for TOA in an Asian population, with high BMI and large TOA size identified as independent risk factors. With our findings of significantly prolonged hospitalization stay and a longer duration of intravenous antibiotics in patients who failed conservative medical management and required invasive intervention, the authors advocate early identification of such patients, in order to ensure institution of prompt and timely invasive intervention with resultant avoidance of prolonged hospitalization, antibiotic usage, and patient morbidity.

## Figures and Tables

**Table 1:** Demographic characteristics and comparison between the two groups.

	All	Medical	Surgical	P-value
	(N=136)	(N=111)	(N=25)	
*Demographics*				

Age (years)				
Mean ± SD	37.7 ± 9.7	37.2 ± 9.8	39.9 ± 9.0	0.209
Q_1_	31	30	36	
Median	38	38	41	
Q_3_	46	45	46	

Ethnicity				0.296
Chinese	46 (33.8%)	37 (33.3%)	9 (36.0%)	
Malay	48 (35.3%)	40 (36.0%)	8 (32.0%)	
Indian	16 (11.8%)	13 (11.7%)	3 (12.0%)	
Others	26 (19.1%)	21 (18.9%)	5 (20.0%)	

Parity				
Mean ± SD	1.4 ± 1.4	1.4 ± 1.4	1.7 ± 1.6	0.278
Q_1_	0	0	0	
Median	1	1	2	
Q_3_	2	2	2	

BMI (kg/m^2^) ^a^				
Mean ± SD	26.5 ± 7.6	25.5 ± 5.4	30.6 ± 12.4	0.011

History of PID				
Yes	20 (14.7%)	16 (14.4%)	4 (16.0%)	0.763
No	116 (85.3%)	95 (85.6%)	21 (84.0%)	

BMI: Body Mass Index.

PID: Pelvic inflammatory disease.

^a^  Missing data for 10 patients.

**Table 2:** Comparison of clinical parameters on admission between the two groups.

	All	Medical	Surgical	P-value
	(N=136)	(N=111)	(N=25)	
*Clinical presentation*				

Pain	131 (96.3%)	107 (96.4%)	24 (96.0%)	0.924
If yes, days of pain (days)	4.9 ± 6.7	5.1 ± 7.2	4.3 ± 3.6	0.278
Mean ± SD				

Fever	66 (48.5%)	44 (39.6%)	22 (88.0%)	<0.001

Vaginal discharge	35 (25.7%)	29 (26.1%)	6 (24.0%)	0.524

Abdominal rebound/guarding	36 (26.5%)	26 (23.4%)	10 (40.0%)	0.090

Adnexal tenderness/cervical motion excitation ^b^	105 (80.2%)	87 (79.8%)	18 (81.8%)	0.830

*Investigations*				

TW count				
Mean ± SD	15.9 ± 5.9	15.5 ± 5.5	18.1 ± 7.2	0.079
<10	18 (13.2%)	15 (13.5%)	3 (12.0%)	0.293
10-20	90 (66.2%)	76 (68.5%)	14 (56.0%)	
>20	28 (20.6%)	20 (18.0%)	8 (32.0%)	

CRP ^c^				0.050
Mean ± SD	157.8 ± 108.2	151.7 ± 112.7	183.7 ± 83.5	0.140
<100	48 (36.6%)	43 (40.6%)	5 (20.0%)	
100-200	41 (31.3%)	32 (30.2%)	9 (36.0%)	
>200	42 (32.1%)	31 (29.2%)	11 (44.0%)	

Positive cervical culture ^d^	23 (17.2%)	22 (20.0%)	1 (4.2%)	0.062

Unilateral/bilateral TOA				
Unilateral	82 (60.3%)	67 (60.4%)	15 (60.0%)	0.973
Bilateral	54 (39.7%)	44 (39.6%)	10 (40.0%)	

Size of TOA (cm)				
Mean ± SD	7.2 ± 3.5	6.9 ± 3.6	8.2 ± 2.7	0.002
<4	11 (8.1%)	10 (9.0%)	1 (4.0%)	0.009
4-8	92 (67.6%)	80 (72.1%)	12 (48.0%)	
>8	33 (24.3%)	21 (18.9%)	12 (48.0%)	

TW count: Total white count.

CRP: C-reactive protein.

TOA: Tubo-ovarian abscess.

^b^ Missing data for 3 patients.

^c^ Missing data for 5 patients.

^d^ Missing data for 2 patients.

**Table 3:** Multivariate adjusted odds ratios of patients who underwent medical and surgical treatment. Each factor in the model is adjusted for all other factors.

Variables	Adjusted Odds ratio	P-value
	(95% Confidence interval)	
Patient age	0.99 (0.93-1.05)	0.700

Ethnicity		
Chinese #	-	0.931
Malay	0.96 (0.20-4.57)	0.961
Indian	0.67 (0.11-3.96)	0.657
Others	0.60 (0.07-5.00)	0.636

CRP on admission	1.00 (1.00-1.01)	0.838

Body mass index	1.10 (1.00-1.21)	0.040

Size of TOA	1.28 (1.03-1.61)	0.030

# Chinese as reference ethnicity.

**Table 4:** Comparison of clinical progress between the two patient groups.

	All	Medical	Surgical	P-value
	(N=136)	(N=111)	(N=25)	
*Clinical progress*				

Days hospitalized				
Mean ± SD	5.7 ± 3.4	4.5 ± 2.0	10.8 ± 3.6	<0.001

Days of IV antibiotics received inpatient				
Mean ± SD	4.6 ± 3.5	3.6 ± 2.2	9.4 ± 4.3	<0.001

Day of IV antibiotics before invasive treatment				-
Mean ± SD	4.0 ± 2.1	NA	4.0 ± 2.1	

## Data Availability

The data used to support the findings of this study are available from the corresponding author upon request.
